# Reliable predictive frameworks for thermal conductivity of ester biofuels using artificial intelligence approaches

**DOI:** 10.1038/s41598-025-19893-9

**Published:** 2025-10-15

**Authors:** Walid Abdelfattah, Ramdevsinh Jhala, Ramachandran Thulasiram, Ahmed Mohsen, Aman Shankhyan, Manoj Kumar Ojha, Dhirendra Nath Thatoi, Fereydoon Ranjbar

**Affiliations:** 1 Department of Mathematics, College of Science, Northern Border University, Arar, Saudi Arabia; 2 Department of Mechanical Engineering, Faculty of Engineering & Technology, Marwadi University Research Center, Marwadi University, Rajkot, 360003 Gujarat India; 3 Department of Mechanical Engineering, School of Engineering and Technology, JAIN (Deemed to be University), Bangalore, Karnataka India; 4 Refrigeration &Air-condition Department, Technical Engineering College, The Islamic University, Najaf, Iraq; 5 Refrigeration &Air-condition Department, Technical Engineering College, the Islamic University of Al Diwaniyah, Al Diwaniyah, Iraq; 6 Centre for Research Impact & Outcome, Chitkara University Institute of Engineering and Technology, Chitkara University, Rajpura, 140401 Punjab India; 7 Department of Mechanical Engineering, Raghu Engineering College, Visakhapatnam, 531162 Andhra Pradesh India; 8 Department of Mechanical Engineering, Siksha ’O’ Anusandhan (Deemed to be University), Bhubaneswar, 751030 Odisha India; 9 Department of Chemistry, Islamic Azad University, Najafabad Branch, Iran

**Keywords:** Ester biofuels, Liquid thermal conductivity (LTC), Machine learning, Intelligent modeling, Correlation, Energy science and technology, Engineering, Mathematics and computing

## Abstract

Ester biofuels have emerged as promising renewable alternatives to fossil fuels due to their environmental compatibility and favorable combustion characteristics. Accurate knowledge of their liquid thermal conductivity (LTC) is essential for optimizing energy systems, engine performance, and thermal modeling applications. However, existing literature lacks generalizable models capable of estimating LTC across diverse ester biofuels and operating conditions. This study addresses this gap by developing robust machine learning models using a comprehensive dataset comprising 1,641 experimental LTC measurements for 22 different ester biofuels under varied pressures and temperatures. Three advanced computational approaches, including Support Vector Machine (SVM), Decision Tree (DT), and Genetic Programming (GP), were employed to predict LTC based on temperature, pressure, critical thermodynamic properties, and molar weight of the biofuels. Among the developed models, the SVM technique exhibited superior predictive performance with a determination coefficient (*R*^*2*^) of 99.53%, and a mean absolute percentage error (MAPE) of 0.60% for the unseen dataset. The GP model, in turn, produced an explicit mathematical correlation with MAPE of 1.16% and R² of 97.94%, offering a reliable and interpretable alternative. The models demonstrated strong agreement with experimental data across a wide operating range and successfully captured the influence of key parameters on LTC. Sensitivity analysis revealed that temperature plays the most fundamental role in controlling LTC. Compared to existing empirical correlations, the proposed intelligent models provide broader applicability and enhanced accuracy for LTC prediction of ester biofuels. These findings contribute to advancing predictive capabilities in biofuel thermal property modeling and support the efficient integration of ester biofuels into energy systems.

**Supplementary Information:**

The online version contains supplementary material available at 10.1038/s41598-025-19893-9.

## Introduction

The global energy landscape remains heavily reliant on the combustion of fossil fuels, which continues to dominate electricity generation and transportation sectors^1^. However, the environmental toll of this dependence is becoming increasingly unsustainable^2,3^. The release of sulfur dioxide contributes to acid rain, while carbon dioxide emissions drive global climate change, posing serious ecological and public health risks. In response, the search for cleaner and more sustainable energy carriers has intensified^4^. Among various alternatives such as hydrogen, liquefied natural gas, and alcohol-based fuels, biofuels have gained significant traction due to their renewable origin and compatibility with existing infrastructure^5^. One class of biofuels, known as ester biofuels, is produced via transesterification, a chemical process that converts triglycerides from vegetable oils or animal fats into short-chain alkyl esters using alcohols^6–11^. These ester biofuels offer numerous environmental and operational advantages over petroleum-based fuels. Their low sulfur content, high biodegradability, reduced greenhouse gas emissions, and improved combustion characteristics position them as viable replacements for conventional diesel^12–16^. Additionally, their physical properties, such as density, viscosity, and flash point, are comparable to those of fossil fuels, allowing their use in modern compression-ignition engines without significant modification^17^. The cleaner burn profile, superior cetane number, and lubricity of ester biofuels further enhance engine longevity and performance^18–20^.

A critical factor in integrating ester biofuels into thermal and combustion systems is a thorough understanding of their thermophysical properties, particularly LTC^21–24^. The LTC of fuels plays a fundamental role in predicting heat transfer performance, optimizing engine combustion, and designing fuel handling systems^25–27^. Accurate LTC values are essential for thermal management, simulation of in-cylinder processes, and evaluation of energy efficiency in biofuel-based engines. Given the diversity of ester biofuels and their sensitivity to operating conditions such as temperature and pressure, developing generalizable and precise LTC models is essential. Despite increasing research interest, reliable predictive tools that can estimate LTC across various ester biofuels remain limited, highlighting the need for advanced modeling strategies^28^.

Over the past decade, numerous experimental efforts have been devoted to measuring the LTC of ester biofuels under varied thermodynamic conditions. These studies provide a valuable foundation for understanding how ester structure, carbon chain length, and operating parameters influence thermal transport properties. Table 1 summarizes key experimental works encompassing a wide range of esters and testing conditions. A number of studies have focused primarily on short- and medium-chain esters at atmospheric pressure, while more recent research expanded to high-pressure regimes and long-chain unsaturated esters. For instance, Perkins and Huber^29^conducted extensive measurements on methyl oleate and methyl linoleate under pressures exceeding 40 MPa. Others, such as Zheng et al^30,31^., explored thermal conductivity in broader temperature-pressure domains, enhancing the diversity and representativeness of the data. Altogether, the literature accounts for 1,641 experimental LTC data points, forming a robust and diverse foundation for developing predictive models that can generalize across a wide spectrum of ester biofuel types and operating conditions.

**Table 1:** Summary of experimental studies on LTC of ester biofuels.

Source	Ester biofuel	Temperature, (K)	Pressure, (MPa)	Number of data
^32^	Ethyl hexanoate, Ethyl pentanoate, Ethyl butyrate, Ethyl propionate, Ethyl acetate	$\:249$ to $\:383$	$\:0.1$	96
^21^	Methyl caprate, Methyl laurate, Methyl myristate	$$\:267$$ to $$\:403$$$$\:K$$	$$\:0.1$$	78
^33^	Methyl pelargonate, Methyl caprylate, Methyl caproate, Methyl pentanoate, Methyl butyrate	$\:263$ to $\:393$	$\:0.1$	99
^22^	Ethyl heptanoate, Ethyl caprylate, Ethyl caprate, Ethyl laurate, Ethyl myristate	$\:250$ to $\:344$	$\:0.1$	77
^26^	Methyl caprate, Methyl laurate, Methyl myristate	$\:291$ to $\:371$	$\:0.1\:$to $\:15$	60
^29^	Methyl Linoleate, Methyl Oleate	$\:301$ to $\:\:509$	$\:0.082\:$to $\:42.75$	745
^30^	Methyl Decanoate, Methyl Octanoate, Methyl Pentanoate	$\:293$ to $\:523$	$\:0.1\:$to $\:15$	328
^31^	Methyl caproate, Methyl butyrate	$\:303$ to $\:523$	$\:0.1\:$to $\:15$	158
**Total**		249 to 523	0.082 to 42.75	**1641**

Despite numerous experimental efforts, only a limited number of empirical models have been proposed to estimate the LTC of ester biofuels. These models typically rely on multi-variable polynomial regressions that correlate LTC with key operational parameters, primarily temperature and/or pressure. A generalized form of such correlations is expressed as:

1 $$\:\lambda\:=\sum\:_{i=0}^{k}\sum\:_{j=0}^{k}{m}_{ij}{T}^{i}{P}^{j}$$

where *k* is the polynomial order, and $\:{m}_{ij}$​ are empirically fitted coefficients derived through regression on experimental data. Although this approach can offer accurate fits within specific data sets, it suffers from critical limitations. Notably, the coefficients $\:{m}_{ij}$​ must be re-calibrated for each individual ester biofuel and each specific range of operating conditions. This restricts the practical utility of such correlations, particularly when modeling diverse fuel types across broad thermodynamic domains. Furthermore, most existing correlations have been developed for a narrow subset of ester biofuels^32,34,35^typically under limited laboratory conditions, and therefore lack generalizability. As a result, traditional polynomial-based methods fall short in providing a universal and scalable predictive tool for LTC estimation in the context of real-world biofuel applications.

Machine learning (ML) techniques offer a powerful data-driven alternative for modeling a wide spectrum of energy and process engineering problems^5,36–42^. These intelligent models are capable of capturing complex, nonlinear interactions among influencing factors such as thermodynamical and molecular characteristics, without relying on predefined equations. Their adaptability enables them to learn directly from experimental trends, making them highly suitable for systems with significant variability across different ester biofuels. Despite their proven success in predicting other fuel properties^14,43^the application of ML tools for LTC estimation in ester biofuels remains largely unexplored. This presents a clear research opportunity to leverage advanced ML algorithms for developing accurate, generalizable LTC prediction models across a broad range of operating conditions and fuel types.

Despite the fundamental importance of LTC in the design and optimization of thermal and combustion systems, existing models for ester biofuels remain highly case-specific and lack generalizability. All correlations reported in the literature are derived from limited experimental datasets, often focusing on a small subset of esters and narrow temperature–pressure ranges. These correlations typically adopt multi-parameter polynomial regressions that require recalibration for each compound and operating condition, which restricts their practical applicability in real-world systems where diverse Fuels and broad operating domains are encountered. As a result, there is a clear research gap in the development of predictive frameworks that can accurately estimate LTC across a wide spectrum of ester biofuels under varied thermodynamic conditions. The present study addresses this gap by developing robust machine learning-based LTC prediction models trained and validated on a comprehensive dataset of 1,641 experimental measurements covering 22 different esters across wide temperature and pressure ranges. Unlike empirical correlations, which are often constrained by their functional form and calibration domain, the proposed models, based on SVM, DTT, and GP, are capable of learning complex nonlinear relationships directly from data. This approach not only enhances predictive accuracy but also provides broader applicability without the need for fuel-specific fitting. Furthermore, the GP technique offers an explicit analytical expression, bridging the gap between data-driven modeling and practical engineering usability. Hence, the main contributions of this work are: (i) the development of accurate and generalizable LTC models applicable to diverse ester biofuels; (ii) the derivation of an interpretable correlation for engineering practice; (iii) a comparative assessment against existing empirical correlations to highlight the advantages of the proposed methods; and (iv) a sensitivity analysis to elucidate the influence of key thermodynamic and molecular descriptors on LTC. Collectively, this integrated framework advances the state of LTC modeling and supports the reliable integration of ester biofuels into energy conversion and thermal management applications.

## Materials and methods

### Artificial intelligence approaches

The selection of machine learning techniques in this study was guided by the need to balance predictive accuracy, interpretability, and computational efficiency. SVM was chosen for its proven capability in modeling highly nonlinear relationships in small-to-medium-sized datasets, making it particularly suitable for capturing the complex dependencies of thermal conductivity on thermodynamic and molecular descriptors. DT was included as a complementary approach due to its transparency, fast training, and ease of interpretation, which are valuable for gaining insight into feature importance and for deployment in resource-constrained environments. GP, on the other hand, was employed to generate explicit analytical correlations through symbolic regression, offering a physically interpretable alternative that can be directly implemented in engineering calculations without requiring specialized ML software. Together, these three methods provide a comprehensive and well-justified modeling framework: SVM ensures high fidelity, DT offers simplicity and interpretability, and GP bridges the gap between data-driven modeling and practical engineering usability.

#### DT

**Fig. 1:**
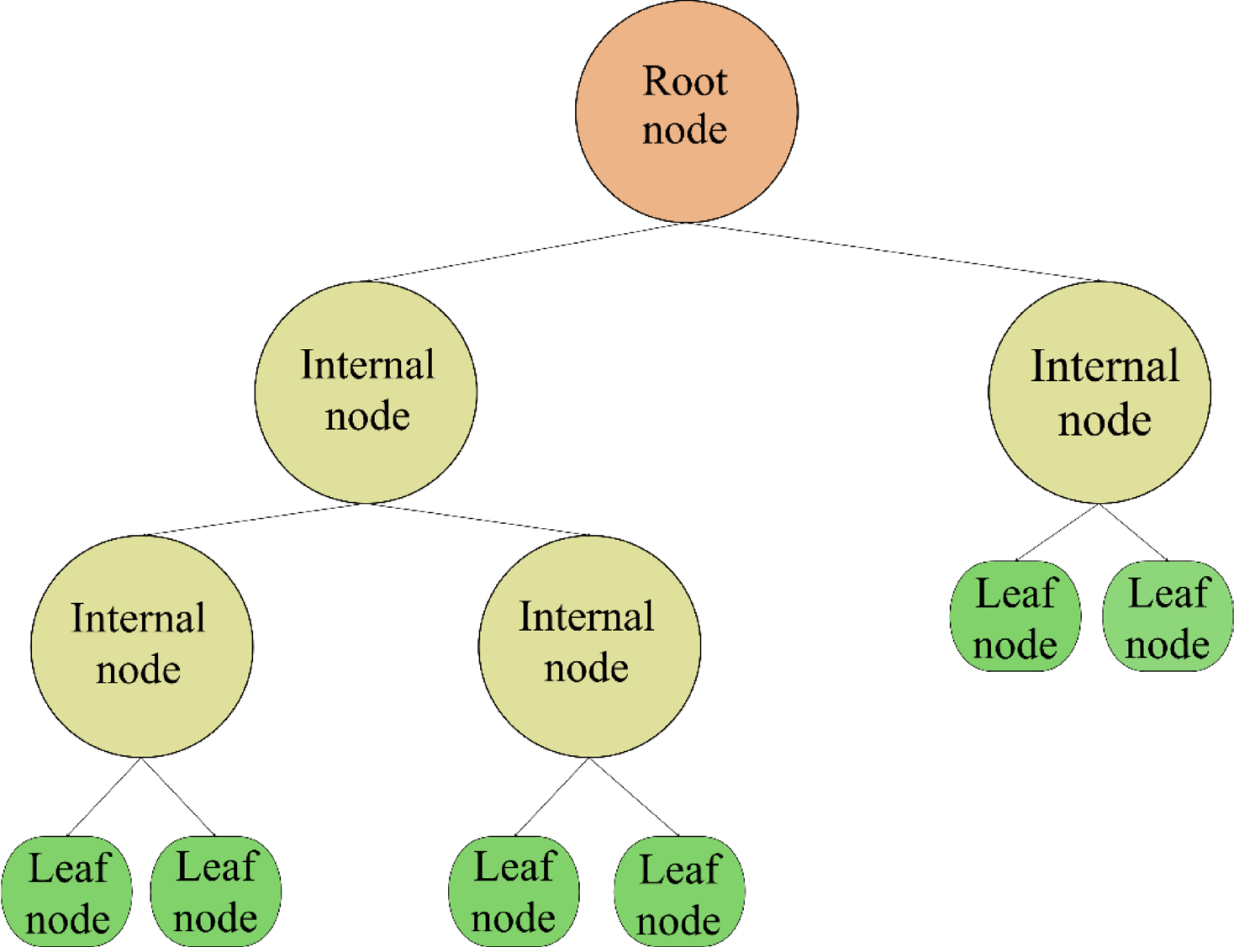
A schematic diagram of the DT model.

Unlike the conventional parametric models, decision tree (DT) is a flexible, non-parametric approach for both modeling and classification problems^44–49^. A regression tree benefits from hierarchical structure, which is similar to a branching diagram, as illustrated in Fig. 1. At the top sits the initial node, encompassing the complete dataset. Subsequent segments, known as internal nodes, have one entry point but diverge into several exit paths. These nodes represent decisions based on specific data features^50–53^. The concluding nodes or leaves, represent the ultimate outcome of the decisions.

The design of a regression tree-based model involves three key phases: data segmentation, stopping, and pruning. Data segmentation, also called splitting, divides the dataset into more homogenous groups. This division depends on pinpointing the most influential data feature for distinction^54,55^. Selection commonly uses indicators such as misclassification cost, the Gini impurity measure, gain of information, and gain ratio, as detailed by Patel and Upadhyay^56^. To prohibit model over-fitting and preserve the ability to generalize, strict stopping conditions are used. These limitations control how complex the decision tree can be, by establishing boundaries on how few data entries can be in a node before further division, and by capping the maximum path length^57^. Without these criteria, the tree may become complicated, flawlessly categorizing the data it was trained on, but performing poorly on new data. When stopping rules alone are inadequate to prevent over-fitting, simplification (or pruning) becomes useful. After initially creating a complete decision tree, irrelevant segments, judged by their minimal contribution to model accuracy, are methodically eliminated^58–60^. This refinement produces a more concise and reliable model, equipped with improved predictive accuracy on unseen data.

#### SVM

Support Vector Machines (SVMs) are a widely used machine learning technique for both regression and classification tasks^61–65^. This approach to machine learning relies on the principle of structural risk minimization (SRM). SRM seeks to minimize potential errors during the learning process, thereby enhancing the model’s predictive capability^66–73^. The central concept behind SVM involves approximating the training data using a linear regression model^74,75^. This is achieved by first mapping the input feature data into a higher-dimensional feature space. Given a training dataset $\:Z=\left\{{x}_{i},{y}_{i}|\:i=\text{1,2},\dots\:,n\right\}$, where $\:{x}_{i}$ represents an *m*-dimensional vector containing the values of the input variables, $\:{y}_{i}$ represents the associated output values, and $\:n$ denotes the total number of training instances, SVM establishes a regression function as follows^76,77^:

2 $$\:y=b+{W}^{T}\theta\:\left(x\right)\:$$

Where $\:b$ signifies the bias, and *W* represents the weight vector^78,79^. The function $\:\theta\:\left(x\right)$ performs a nonlinear transformation, mapping the input *x* into the higher-dimensional space^80^. The optimal values for *W* are determined by minimizing the following objective function:

3 $$\:H=\frac{1}{2}{W}^{2}+C\sum\:_{i=1}^{n}\left({\zeta\:}_{i}+{\zeta\:}_{i}^{*}\right)\:$$

Subject to the following constraints^81^,

4 $$\:{y}_{i}-\left\{b+{W}^{T}\theta\:\left({x}_{i}\right)\right\}\le\:{\zeta\:}_{i}+\psi\:\:\:\:\:\:\:\:\:\:\:\:\:\:\:\:\:\:\:i=\text{1,2},\dots\:,n$$

5 $$\:\left\{b+{W}^{T}\theta\:\left({x}_{i}\right)\right\}\le\:{\zeta\:}_{i}^{*}+\psi\:\:\:\:\:\:\:\:\:\:\:\:\:\:\:\:\:\:\:\:\:\:\:\:\:\:\:\:i=\text{1,2},\dots\:,n$$

Where $\:{\zeta\:}_{i}$ and $\:{\zeta\:}_{i}^{*}$ are slack variables, and $\:\psi\:$ signifies the acceptable error margin during the training phase^82,83^. Furthermore, the parameter $\:C$, known as the penalty factor, governs the balance between the model’s complexity and its agreement with the training data (training deviation)^84^. After defining the constraints and implementing Lagrange multipliers, the ultimate SVM solution is represented by

6 $$\:f\left(x\right)=\sum\:_{i=1}^{n}\left({\mu\:}_{i}-{\mu\:}_{i}^{*}\right)G\left({x}_{i},{x}_{j}\right)+b$$

Where $\:{\mu\:}_{i}$ and $\:{\mu\:}_{i}^{*}$ represent non-negative Lagrange multipliers, and $\:G\left({x}_{i},{x}_{j}\right)$ stands for the kernel function. The performance of an SVM model is highly dependent on the choice of kernel function. In the present investigation, a Gaussian kernel function is utilized,

7 $$\:G\left({x}_{i},{x}_{j}\right)={exp}\left(-\left|\left|x-{x}_{i}\right|\right|/{\sigma\:}^{2}\right)$$

Where $\:\sigma\:$ denotes the variance of the Gaussian function, and is optimized via a tuning process in the model training.

#### GP

**Fig. 2:**
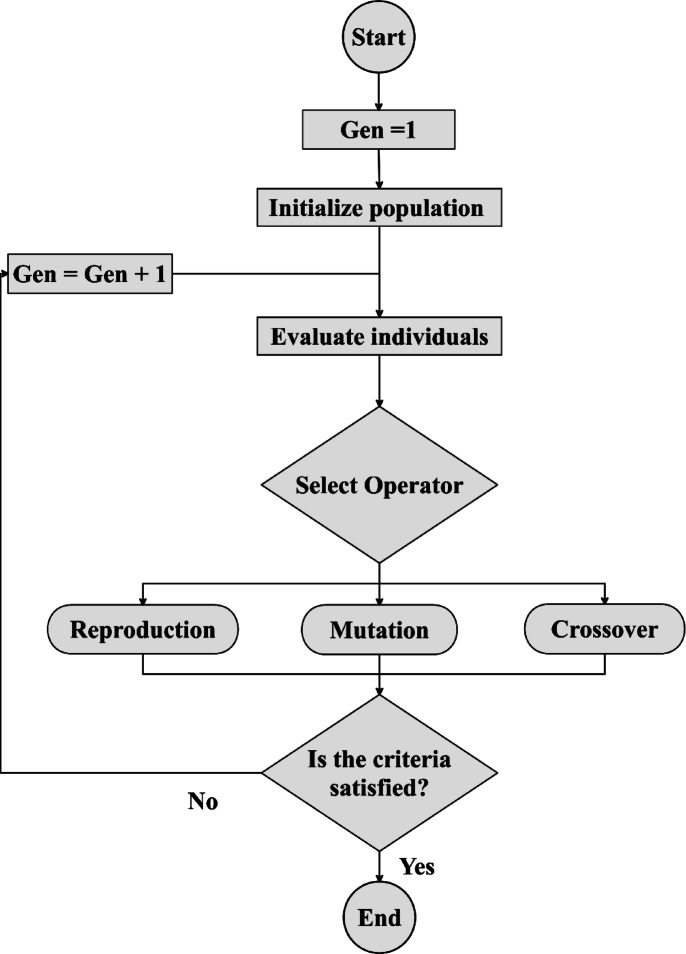
Flowchart of the GP method.

GP is a symbolic regression technique rooted in the principles of natural evolution and population-based optimization. It is particularly valuable for discovering mathematical expressions that define complex, nonlinear relationships between inputs and outputs. In contrast to traditional regression methods that require a predefined functional form, GP evolves analytical equations dynamically through iterative search and adaptation, as shown in Fig. 2, making it ideal for modeling physical phenomena like LTC. The GP process begins with the random generation of an initial population, where each individual, referred to as a chromosome, represents a candidate mathematical expression constructed from a set of operators (e.g., +, –, ×, ÷) and variables (e.g., temperature, pressure, fuel characteristics). The predictive performance of each expression is then evaluated using error metrics, such as MAPE and *R*^*2*^, comparing model outputs with experimental LTC data. To improve the population, GP employs biologically inspired genetic operators:

- Crossover combines segments from two parent equations to generate new offspring with potentially improved structure and accuracy.
- Mutation introduces random changes to parts of an equation, helping maintain diversity and preventing premature convergence on suboptimal solutions.

Through successive generations, the population is refined by selecting high-performing expressions while discarding less effective ones. This evolutionary cycle continues until a termination criterion is met, typically based on reaching a defined accuracy threshold or a maximum number of generations. In the context of this study, GP is used to derive an explicit mathematical correlation for LTC that balances predictive accuracy with interpretability, providing a user-friendly alternative to more complex machine learning models.

### Data gathering

The reliability of any data-driven modeling effort is fundamentally dependent on the quality and comprehensiveness of the underlying dataset. To ensure the development of accurate LTC prediction models, this study compiled a large and diverse set of experimental measurements from peer-reviewed literature summarized in Table 1. In total, 1,641 data points were collected, representing the LTC values of 22 distinct ester biofuels measured under a broad range of temperatures and pressures. The dataset spans operating temperatures from 249 K to 523 K and pressures up to 42.75 MPa, capturing the thermophysical behavior of both methyl and ethyl esters under realistic engine and process conditions. The selected sources maintain a reported experimental uncertainty below 2%, indicating a high level of measurement precision and consistency across studies. This comprehensive and high-fidelity database forms a robust foundation for training and validating the proposed machine learning models, enabling the development of predictive tools with strong generalization capabilities across diverse ester biofuels and operating environments.

### Selection of input features

Accurate prediction of LTC requires careful consideration of the parameters that govern heat transfer behavior in ester biofuels. Experimental studies consistently identify temperature and pressure as the most immediate external factors affecting thermal conductivity, as they directly alter fluid density, molecular interactions, and vibrational energy transfer. Consequently, these two variables were included as core operational inputs in the present models. To capture molecular-level variability among different ester biofuels, additional descriptors were incorporated: critical temperature ($\:{T}_{c}$), critical pressure ($\:{P}_{c}$), and molecular weight ($\:Mw$). These parameters are widely recognized as fundamental thermophysical properties that encode intermolecular cohesion, volatility, and structural complexity. Incorporating them enables the models to generalize across diverse ester compounds without requiring compound-specific fitting. The resulting formulation can be expressed as:

8 $$\:\lambda\:=f\left(P,\:T,{P}_{c},{T}_{c},Mw\right)$$

This multidimensional input set ensures the models are equipped to capture both operational and molecular influences on LTC with high fidelity.

This multidimensional feature set balances operational conditions ($\:P,\:T$) with molecular descriptors ($\:{P}_{c},{T}_{c},Mw$), ensuring that both environmental effects and intrinsic molecular characteristics are considered. While other potential descriptors, such as acentric factor, polarity indices, or group-contribution parameters, could provide additional granularity, such data are not consistently available across the broad experimental dataset compiled here. The selected features therefore represent a practical and physically meaningful compromise between data availability, predictive fidelity, and generalizability, making them appropriate for robust LTC modeling of ester biofuels.

To demonstrate the scope and applicability of the developed predictive models, the statistical characteristics of the input variables used for model training and validation are summarized in Table 2. The data set spans a broad thermodynamic and molecular domain, ensuring that the models are exposed to highly diverse operating conditions and ester structures. The operating pressure ranges from near-atmospheric levels to over 42 MPa, covering both standard engine conditions and elevated pressures relevant to advanced combustion systems and process equipment. Similarly, the temperature extends from sub-ambient values around 249 K up to 523 K, encompassing cold-start scenarios as well as elevated thermal environments typical of engine and industrial applications. The molecular descriptors also exhibit wide variation, reflecting the diversity of ester biofuels considered. Molecular weights extend from less than 90 g/mol, representative of short-chain esters, up to nearly 300 g/mol, corresponding to long-chain compounds with more complex structures. Likewise, the critical temperature and critical pressure values cover a broad spectrum, capturing both volatile, lighter esters and more stable, heavier counterparts. The relatively large standard deviations observed across all parameters highlight the heterogeneity of the dataset, which is critical for building models with strong generalization capability. Instead of being tailored to a narrow set of compounds or operating conditions, the developed frameworks are grounded in a database that reflects the true diversity of ester biofuels and their application environments. This broad coverage not only enhances the reliability of the predictions but also ensures that the models remain applicable across a wide range of real-world scenarios, from engine simulations to thermal system design.

**Table 2:** Range and variability of input features defining the applicability domain of the developed models.

Parameter	Unit	Minimum	Maximum	Average	Median	Standard deviation
$\:P$	MPa	0.082	42.75	10.26	5	12.73
$\:T$	K	249.13	523.68	383.67	363.61	72.29
$\:{P}_{c}$	MPa	1.20	4	1.94	1.71	0.83
$\:{T}_{c}$	K	530	896.01	726.06	720.05	116.49
$\:Mw$	gr/mol	88.11	296.49	218.99	214.34	76.61

### Statistical evaluation

To quantify the predictive performance of the developed models for LTC estimation in ester biofuels, several statistical error metrics were employed. These metrics provide insight into the models’ accuracy, consistency, and reliability when compared to experimental data.

MAPE evaluates the average magnitude of relative errors across all predictions:

9 $$\:MAPE\:\left(\%\right)=\frac{1}{N}\sum\:\left|{E}_{i}\right|\times\:100$$

SD of the relative error assesses the spread or variability of prediction errors:

10 $$\:SD\:\left(\%\right)=\sqrt{\frac{\sum\:{\left({E}_{i}-\overline{{E}_{i}}\right)}^{2}}{N-1}}\times\:100$$

RRMSE expresses the root mean square deviation between predicted and actual LTC values, normalized by the average of the experimental values:

11 $$\:RRMSE\:\left(\%\right)=\frac{\sqrt{\frac{1}{N}\sum\:{\left({k}_{pre}-{k}_{exp}\right)}^{2}}}{\frac{1}{N}\sum\:{k}_{exp}}\times\:100$$

The *R*^*2*^ value measures the proportion of variance in the experimental data explained by the model:

12 $$\:{R}^{2}\:\left(\%\right)=\left(1-\frac{{\sum\:\left({k}_{pre}-{k}_{exp}\right)}^{2}}{\sum\:{\left({k}_{exp}-\stackrel{-}{{k}_{exp}}\right)}^{2}}\right)\times\:100$$

Here, the relative error $\:{E}_{i}$​ for each prediction is calculated as:

13 $$\:{E}_{i}=\frac{{k}_{pre,i}-{k}_{exp,i}}{{k}_{exp,i}}$$

By jointly considering MAPE (average accuracy), SD (error variability), RRMSE (scale-normalized deviation), and *R*^*2*^ (variance explained), this framework ensures a robust and multi-dimensional assessment of model performance. Such a combination not only demonstrates predictive accuracy but also guards against misleading interpretations that could arise from relying on a single metric. This comprehensive evaluation therefore provides sufficient validation of the developed models for practical implementation.

### Code availability

The trained machine learning models (DT and SVM) are provided as Supplementary Data S1. An instruction file is included in the archive to guide users on applying these models for calculating the LTC of ester biofuels. These files can be executed in MATLAB R2024a or later. All machine learning models were developed using the MATLAB Regression Learner Toolbox (MATLAB R2024a, The MathWorks Inc., Natick, MA, USA; https://www.mathworks.com/products/regression-learner.html).

## Results and discussions

### Development of the smart predictive tools for LTC prediction

To establish accurate and generalizable models for LTC prediction, two widely used machine learning algorithms, i.e., SVM and DT, were employed. These algorithms were trained to map the relationship between LTC and key variables, as defined in Eq. (8). The dataset was randomly partitioned into training and testing subsets, with 80% of the data (1,314 samples) used to train the models and the remaining 20% (327 samples) reserved for testing and validation. This split ensured that the models were exposed to a diverse yet balanced set of inputs during learning while maintaining unseen data for objective evaluation.

**Fig. 3:**
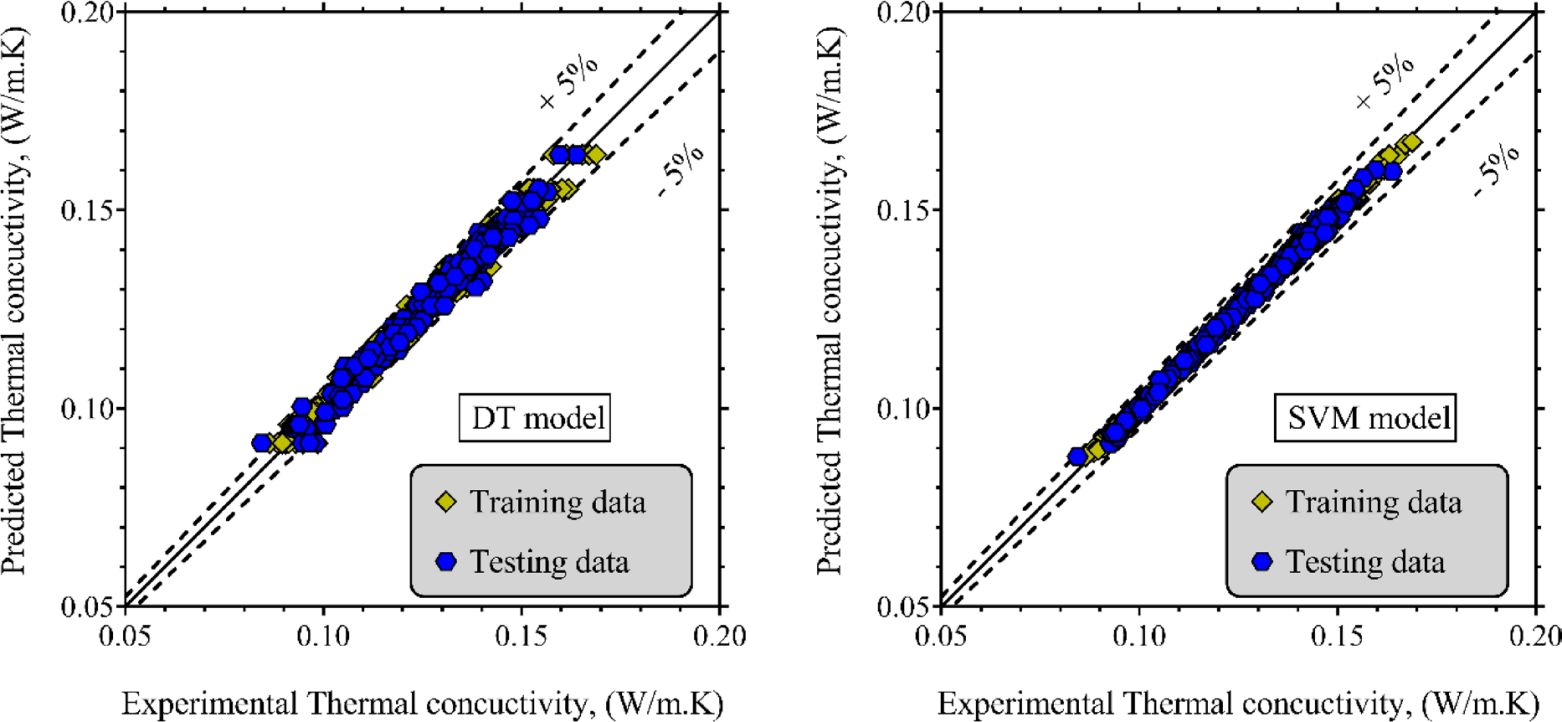
Comparison between experimentally measured and ML-predicted LTC values using SVM and DT models.

Table 3 presents a detailed summary of the statistical performance indicators for both models. The SVM model demonstrates outstanding predictive capability, with *R*^*2*^, RRMSE, SD, and MAPE values being 99.53%, 0.73%, 0.75%, and 0.60%, respectively, on the testing data. These metrics highlight its strong accuracy and generalization capacity across a wide variety of ester biofuels and operational scenarios. On the training set, the model exhibits equally impressive performance, with a MAPE of 0.59% and *R*^*2*^ of 99.54%, indicating minimal overfitting. The DT model also yields promising results, with a MAPE of 1.18% and *R*^*2*^ of 98.12% on the testing data. While slightly less precise than SVM, it remains a reliable and interpretable tool for LTC estimation, particularly in applications where model transparency is preferred. To visualize the predictive performance, Fig. 3 illustrates cross-plots of predicted versus experimental LTC values for both models. The data points are predominantly clustered along the identity line, and most predictions fall within ± 5% error margins. This is particularly evident in the case of the SVM model, whose predictions consistently fall within this narrow band, confirming its robustness and accuracy. Overall, both SVM and DT provide valid frameworks for LTC prediction. However, due to its lower error margins and tighter agreement with experimental measurements, the SVM model can be considered the more reliable and accurate solution for practical applications.

**Table 3:** Accuracy analysis of the novel LTC models.

ML method	Data	$\:{R}^{2}$, (%)	RRMSE, (%)	SD, (%)	MAPE, (%)
DT	Training subset	`99.16	0.97	1.04	0.74
	Testing subset	98.12	1.46	1.61	1.18
SVM	Training subset	99.54	0.71	0.72	0.59
	Testing subset	99.53	0.73	0.75	0.60

To complement the machine learning models and offer an interpretable analytical tool, the GP methodology was implemented for constructing an explicit model to estimate the LTC of ester biofuels. Based on the input features defined in Sect. 2.3, the GP algorithm iteratively evolved symbolic expressions until a high-fidelity equation was obtained that minimized deviation from experimental data. The resulting correlation, shown below, captures the combined effects of key properties:

14 $$\begin{aligned}\:&k=0.189+1.23\times\:{10}^{-4}P\times\:{P}_{c}+1.744\times\:{0}^{-6}P\times\:T+0.0027{P}_{c}^{2}\\&-7.31\times\:{10}^{-5}T-4.58\times\:{10}^{-4}P-2.31\times\:{10}^{-8}Mw\times\:{P}_{c}-5.83\times\:{10}^{-5}T\times\:{P}_{c}\end{aligned}$$

**Fig. 4:**
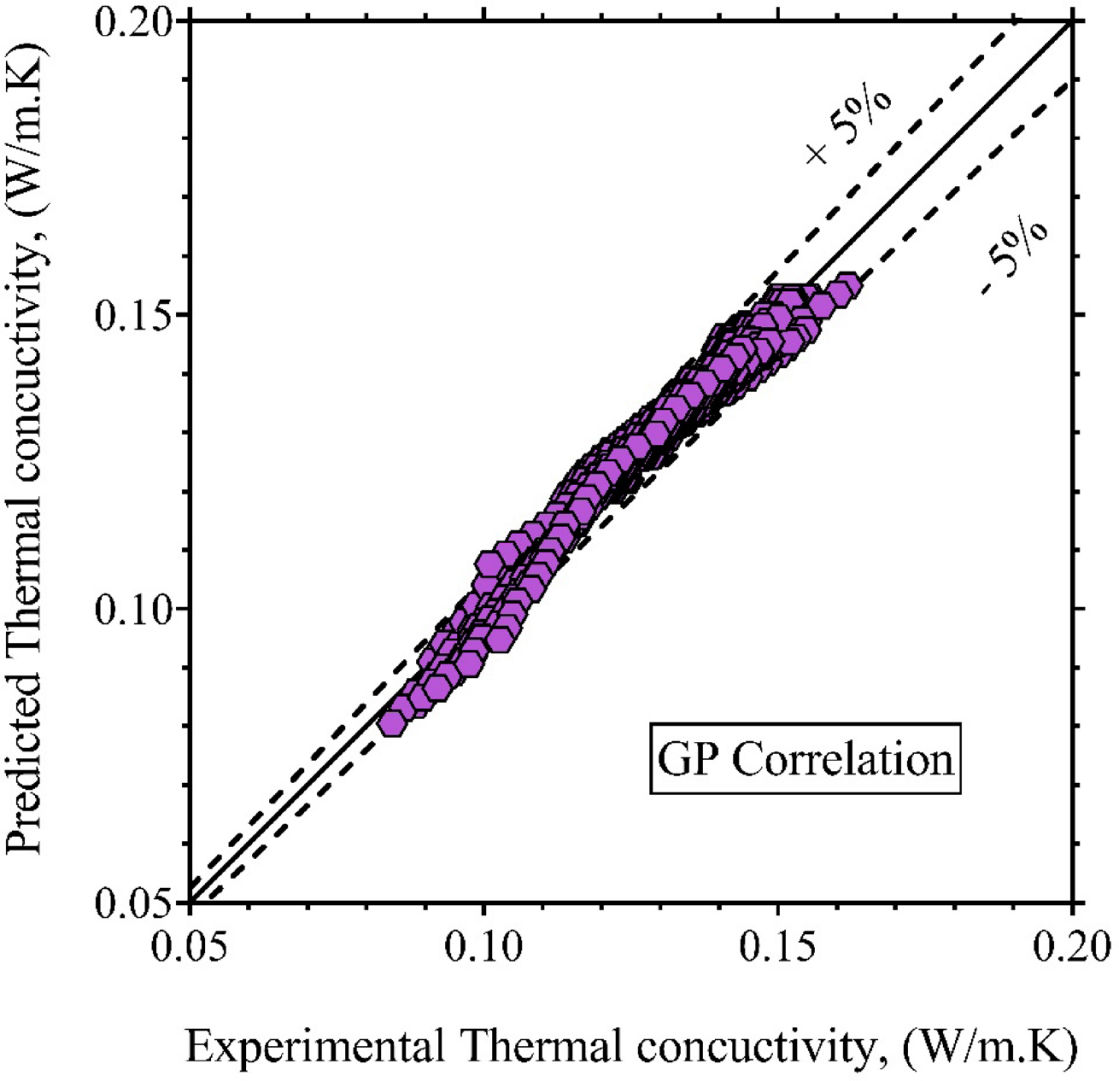
Comparison between experimentally measured and GP-predicted LTC values.

A comparison between the LTC values predicted by Eq. (14) and corresponding experimental measurements is presented in Fig. 4. The correlation exhibits a strong match with the reference data across the full range of conditions, confirming its validity. Statistical evaluation of this expression yielded *R*^*2*^, RRMSE, SD and MAPE of 97.94%, 1.52%, 1.59%, and 1.16%, respectively. These results demonstrate that the GP-derived correlation provides a reliable and computationally efficient method for estimating LTC in a variety of ester biofuels, making it a practical tool for engineering analysis and simulation workflows.

Assessing the generalization ability of predictive models is a crucial step to ensure that the achieved performance is not simply the result of favorable data partitioning. While the initial evaluation relied on an 80/20 train–test split, this approach alone may not fully capture the model’s robustness across all possible data divisions. To address this limitation and rigorously evaluate predictive stability, a five-fold cross-validation (CV) procedure was conducted based on the SVM model, as the most accurate predictive tool provided in this study. This technique provides a more comprehensive assessment of model generalizability by ensuring that each data point is used for both training and testing in different folds. In the CV procedure, the complete dataset was randomly divided into five equal subsets. For each iteration, four subsets (80% of the data) were used to train the SVM model, while the remaining subset (20%) was reserved for testing. This process was repeated five times such that every fold served once as the testing subset, and the fold-wise performance was recorded. The obtained results, summarized in Table 4, confirm the high stability and robustness of the developed SVM framework. Training errors remains tightly bounded between 0.58% and 0.61%, while testing errors varied only slightly between 0.59% and 0.65%. The close similarity of training and testing MAPEs indicates that the model is not overfitting and can reliably capture the underlying physical relationships governing liquid thermal conductivity. Moreover, the narrow dispersion of errors across folds demonstrates that predictive accuracy is not dependent on a particular data partition, highlighting the strong generalizability of the SVM model. This outcome provides further confidence in the reliability of the proposed framework for practical applications in energy system design and biofuel process simulations.

**Table 4:** Five-fold cross-validation performance of the SVM model for predicting LTC of ester biofuels.

	The fold used as the testing subset				
	Fold 1	Fold 2	Fold 3	Fold 4	Fold 5
MAPE (Training), %	0.61	0.60	0.59	0.60	0.58
MAPE (Testing), %	0.62	0.62	0.60	0.59	0.65

### Evaluation of model stability and predictive confidence

Beyond high average accuracy, a robust predictive model must also demonstrate consistency, low dispersion in error, and resilience to variations in input data. To rigorously assess the reliability of the proposed LTC models (SVM, DT, and GP), two complementary statistical tools were used: box plots for visualizing error distributions, and cumulative frequency curves for evaluating prediction confidence across varying accuracy thresholds.

#### Error variability assessment using box plots

**Fig. 5:**
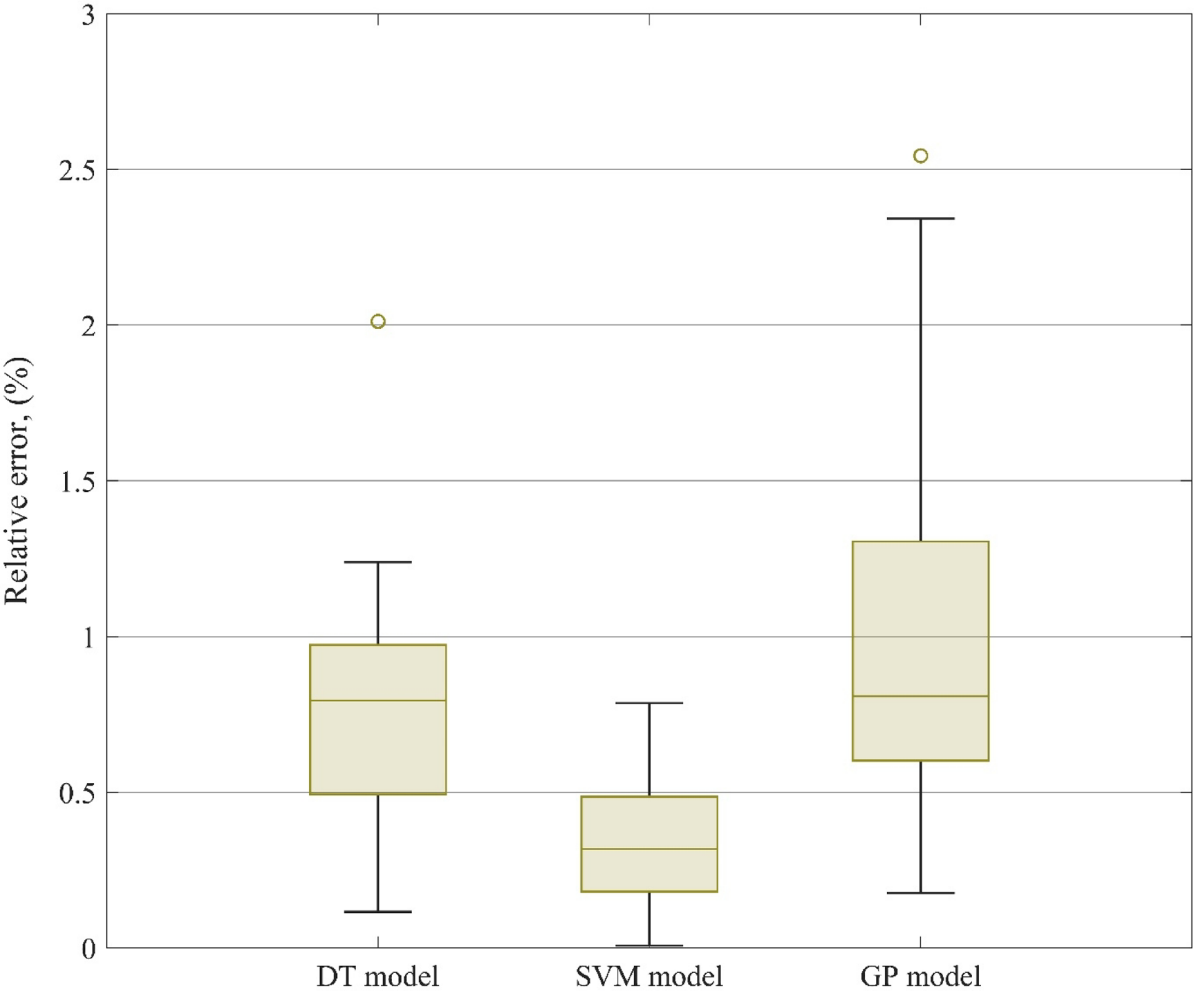
Distribution of absolute relative errors for the SVM, DT, and GP models shown using box plots.

Figure 5 illustrates the distribution of absolute relative errors associated with each model using box-and-whisker plots. These plots offer a compact statistical summary of how prediction errors are spread across the dataset, helping to assess the stability and central tendency of each model’s output. The SVM model clearly exhibits the tightest and most symmetric error distribution, with a median absolute error of 0.32% and an interquartile range (IQR) of just 0.18% to 0.49%. This indicates that more than half of the predictions lie within a very narrow error band, highlighting the model’s ability to deliver consistently accurate results across a broad range of input conditions and ester biofuels. In comparison, the DT model has a slightly wider IQR, spanning from 0.49% to 0.97%, with a median error of 0.80%. While slightly less precise than SVM, the DT model still maintains relatively compact dispersion, suggesting it remains a dependable alternative where computational simplicity or interpretability is prioritized. The GP-derived correlation, while not matching the predictive tightness of SVM, still performs well. Its IQR spans from 0.43% to 1.60%, with a moderate number of outliers. Importantly, all three models exhibit maximum relative errors below 3%, even for extreme data points, underscoring the robustness of the overall modeling framework. From an engineering perspective, such low error margins are particularly valuable for applications in combustion modeling, heat exchanger design, or simulation-based optimization, where LTC variations can significantly impact system performance.

#### Cumulative frequency evaluation of prediction errors

**Fig. 6:**
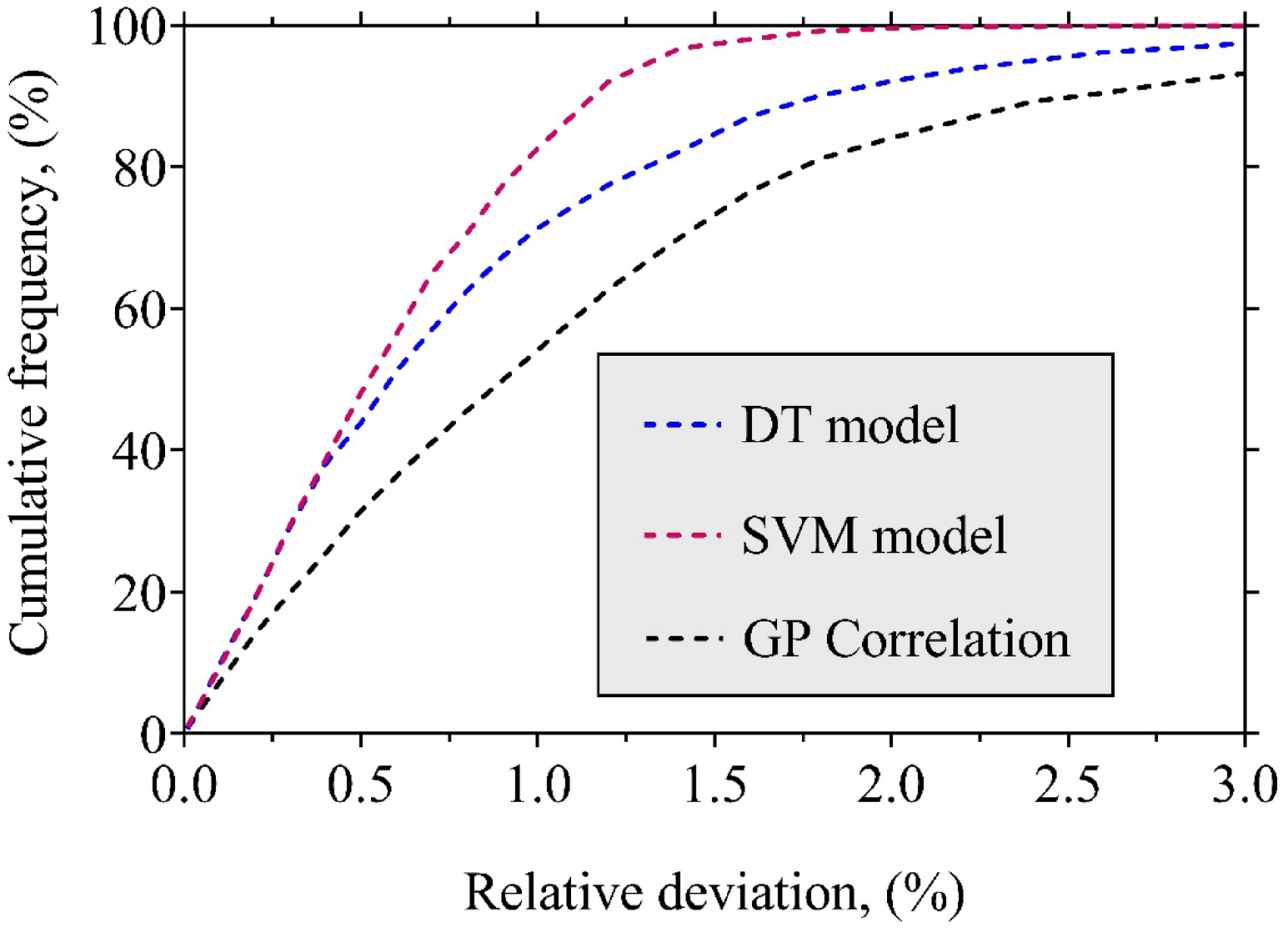
Cumulative percentage of data points predicted within specific error thresholds for the established models.

To further assess and compare model reliability, cumulative frequency curves were constructed and are presented in Fig. 6. These curves show the percentage of predictions falling below specific relative error thresholds, providing a clear visual benchmark for model confidence. The SVM model again demonstrates outstanding performance, with approximately 48.1% of predictions within 0.5% error, 82.6% within 1.0%, 97.3% within 1.5%, and 99.6% within 2.0%. This rapid rise in cumulative frequency at low error thresholds reflects SVM’s ability to capture highly nonlinear interactions among pressure, temperature, and molecular descriptors, enabling it to deliver consistently precise LTC predictions for the vast majority of data points. The kernel-based structure of SVM allows effective mapping of input variables into a higher-dimensional feature space, where linear relationships can be identified and generalized. This gives SVM a distinct advantage when modeling complex thermophysical behavior that cannot be adequately described by simple polynomial or tree-based partitions.

The DT model, though slightly less accurate, still performs reliably, with about 44% of predictions within 0.5%, around 71% within 1%, and 92% within 2%. Its hierarchical splitting strategy makes it interpretable and computationally inexpensive, which is an attractive feature for rapid predictions. However, DT is prone to sensitivity in data partitioning, and small fluctuations in the training set can lead to significant changes in the tree structure. While pruning strategies are employed to mitigate overfitting, the stepwise, piecewise-constant nature of DT predictions limits its ability to capture smooth and continuous nonlinear trends in LTC. This explains its wider error distribution compared to SVM, despite its overall acceptable performance.

The GP-derived correlation, while not competitive at the very lowest error thresholds, still captures over 84% of LTC values within a 2% margin. Its strength lies in producing an explicit, interpretable mathematical equation that engineers can directly apply in design and simulation tasks without needing specialized ML software. Furthermore, its computational cost during application is negligible, since once derived, the correlation reduces to a closed-form expression. The main limitations of GP are its stochastic evolutionary process, which makes training computationally more intensive than DT or SVM, and its tendency to converge to equations that balance accuracy with simplicity rather than maximizing predictive precision. Consequently, GP sacrifices some accuracy for the sake of interpretability and general usability.

Taken together, these results confirm that all three models demonstrate strong reliability and practical value. However, SVM consistently provides the tightest and most accurate predictions by effectively modeling nonlinear, high-dimensional interactions without overfitting. DT offers speed and interpretability but struggles with smooth generalization, while GP offers analytical transparency and lightweight deployment at the expense of slightly higher errors. The complementary nature of these approaches highlights that while SVM is the most suitable choice for high-fidelity LTC estimation, DT and GP remain valuable alternatives in scenarios prioritizing interpretability, simplicity, or minimal computational cost.

### Model performance across different ester biofuel classes

**Fig. 7:**
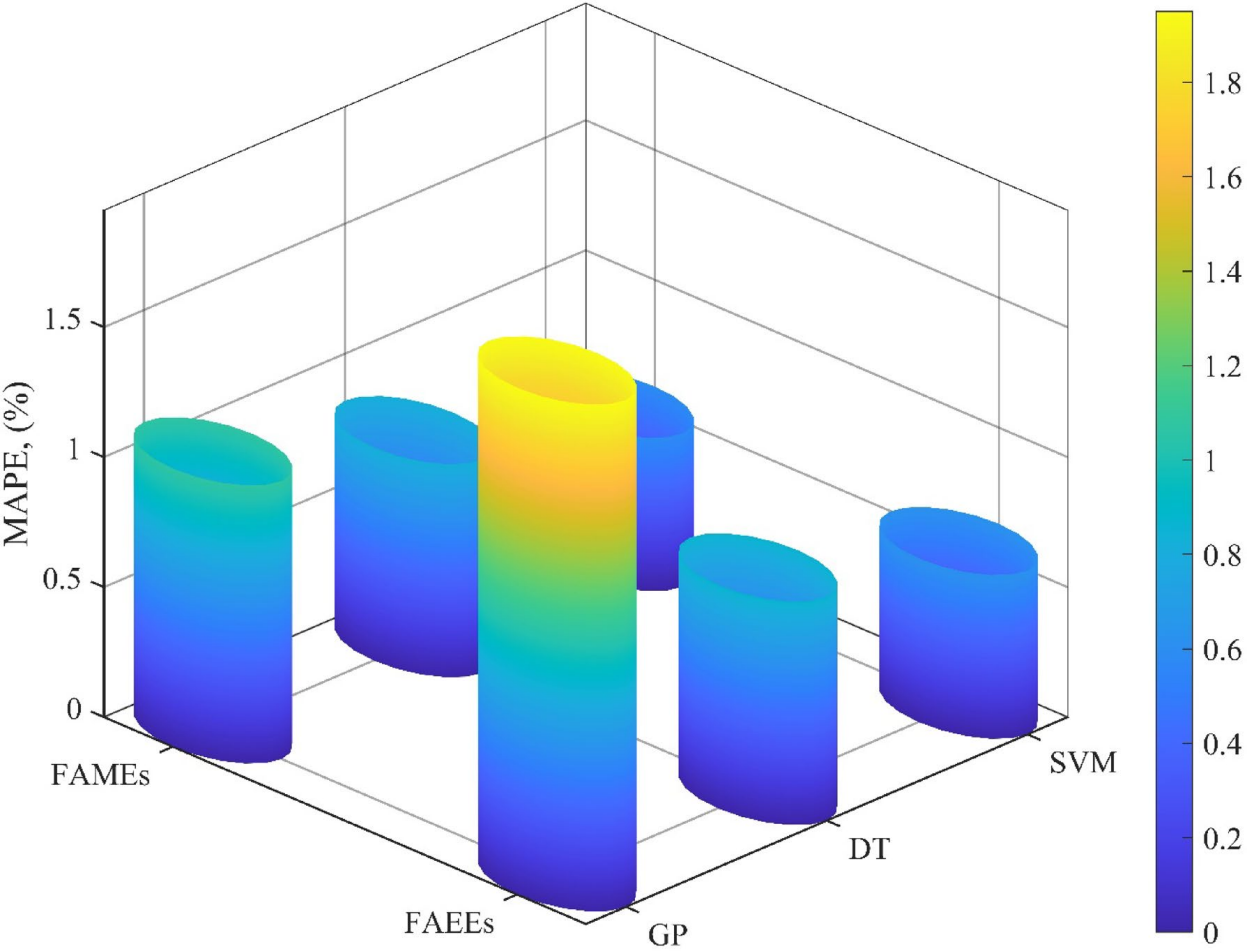
Comparison of prediction accuracy for SVM, DT, and GP models across ester biofuel types.

The assembled LTC dataset (Table 1) encompasses two major categories of ester biofuels: fatty acid methyl esters (FAMEs) and fatty acid ethyl esters (FAEEs). Evaluating model accuracy within each subclass is essential to ensure predictive reliability across structurally diverse fuels. Figure 7 presents a comparative analysis of the MAPE values for each modeling approach when applied separately to FAMEs and FAEEs. The results confirm that all three models maintain a high level of accuracy across both categories, with no significant loss in performance due to molecular variation. Among the models, SVM consistently delivers the highest predictive precision, achieving MAPEs of 0.59% for FAMEs and 0.63% for FAEEs. This negligible difference in error highlights the model’s strong generalization capability across different ester types. The DT model follows, yielding slightly higher MAPEs of 0.82% for FAMEs and 0.86% for FAEEs, while still remaining well within acceptable error bounds for engineering applications. The GP-derived correlation, though slightly less precise, also performs robustly, producing MAPE values below 2% for both ester classes. This demonstrates its applicability as a simplified alternative when an analytical form is preferred for rapid computations or integration into larger simulation environments. The consistent performance across both FAMEs and FAEEs can be attributed to the inclusion of key molecular descriptors, such as molecular weight and critical thermodynamic properties, in the model inputs. These variables enable the models to distinguish between ester types without relying on categorical classification. In summary, the results demonstrate that the proposed approaches, particularly the SVM model, are capable of accurately and reliably estimating LTC across a broad range of ester biofuels, regardless of their chemical structure.

### Outlier detection and influence assessment

In data-driven modeling, ensuring the reliability of predictions requires careful identification of anomalous data points that deviate significantly from general trends. These so-called outliers may originate from experimental inaccuracies, sensor malfunctions, or rare but valid physical phenomena. Regardless of origin, their presence can disproportionately influence model training, degrade accuracy, and reduce generalization capability. To evaluate the sensitivity of the developed LTC models to such data points, an outlier analysis was performed using the William’s plot, a diagnostic tool that combines information from standardized residuals and leverage values. This bivariate plot enables the classification of each sample into three categories:

(i) Acceptable (valid) points with moderate residuals and low influence ($\:\left|SR\right|\le\:3$ and $\:H\le\:{H}^{*}$).
(ii) High-leverage points with structurally influential features ($\:\left|SR\right|\le\:3$ and $\:H>{H}^{*}$).
(iii) True outliers with unusually large prediction errors ($\:\left|SR\right|>3$).

Here, $\:SR$ refers to the standardized residual, and $\:H$ is the leverage score derived from the model’s hat matrix. The warning threshold for leverage, $\:{H}^{*}$, is calculated as:

15 $$\:{H}^{*}=\frac{3\left(X+1\right)}{Y}$$

where $\:X$ is the number of model predictor and $\:Y$ is the total quantity of samples.

**Fig. 8:**
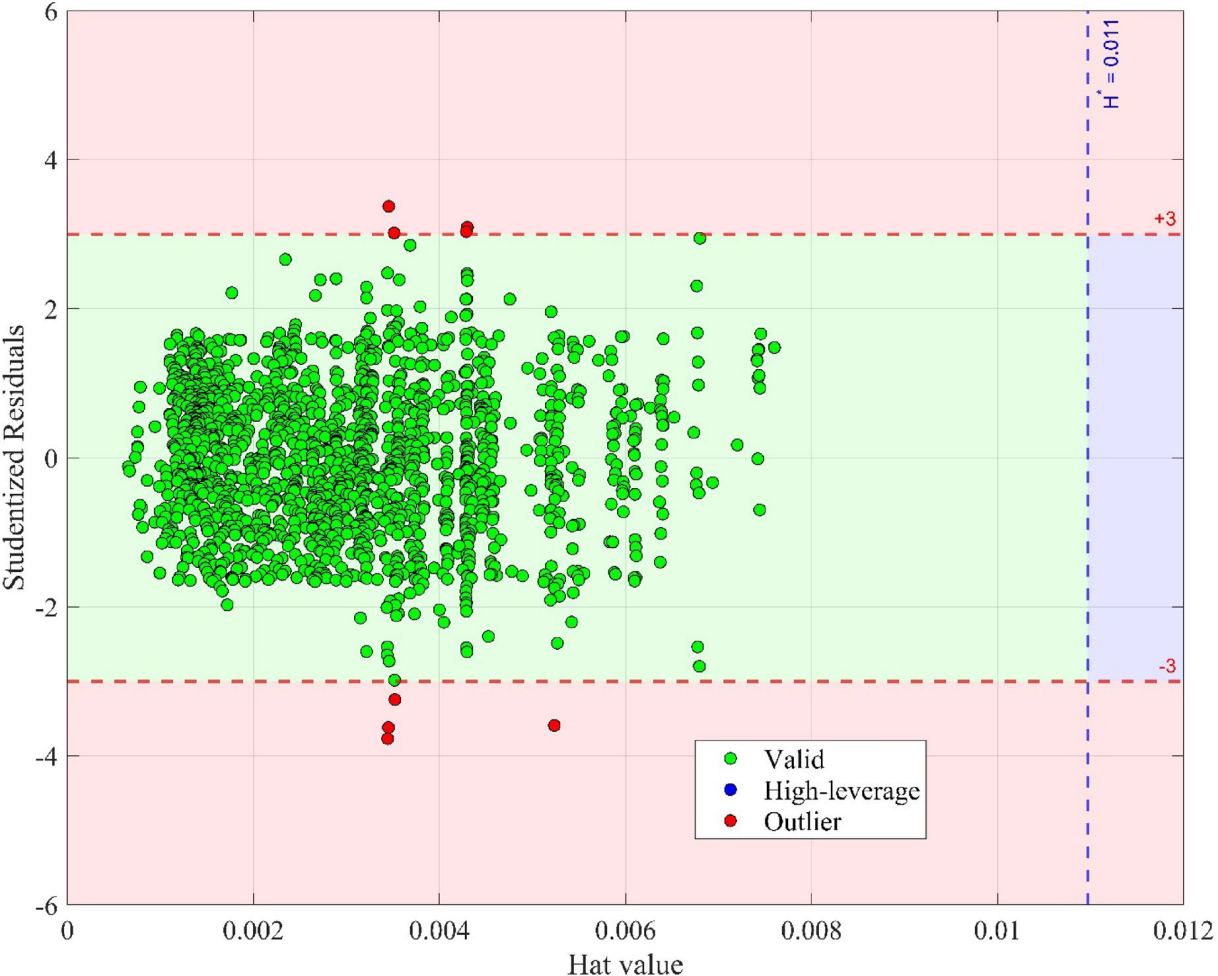
Detection of the suspected data instances based on the William’s plot.

Figure 8 illustrates the William’s plot applied to the SVM model, which exhibited the best overall predictive performance. The plot reveals that the overwhelming majority of data points, over 99% of the 1,641 records, fall within the acceptable region, indicating excellent model stability and a high-quality dataset. Only 8 points are identified as statistical outliers based on their residual magnitude. These represent less than 0.5% of the dataset and are unlikely to have materially influenced the model’s learning or predictive behavior. Importantly, the small number of outliers and the absence of a large cluster of high-leverage points support the robustness and reliability of the proposed models. The LTC prediction framework can thus be applied with confidence across a wide range of ester biofuel types and operating conditions without risk of distortion from atypical samples.

### Influence of thermodynamic and molecular parameters on LTC behavior

To evaluate the physical interpretability of the developed LTC models, a detailed analysis was conducted using the most accurate model, SVM. This investigation explores how LTC responds to changes in key variables, including pressure, temperature, and molecular structure, using representative ester biofuels.

**Fig. 9:**
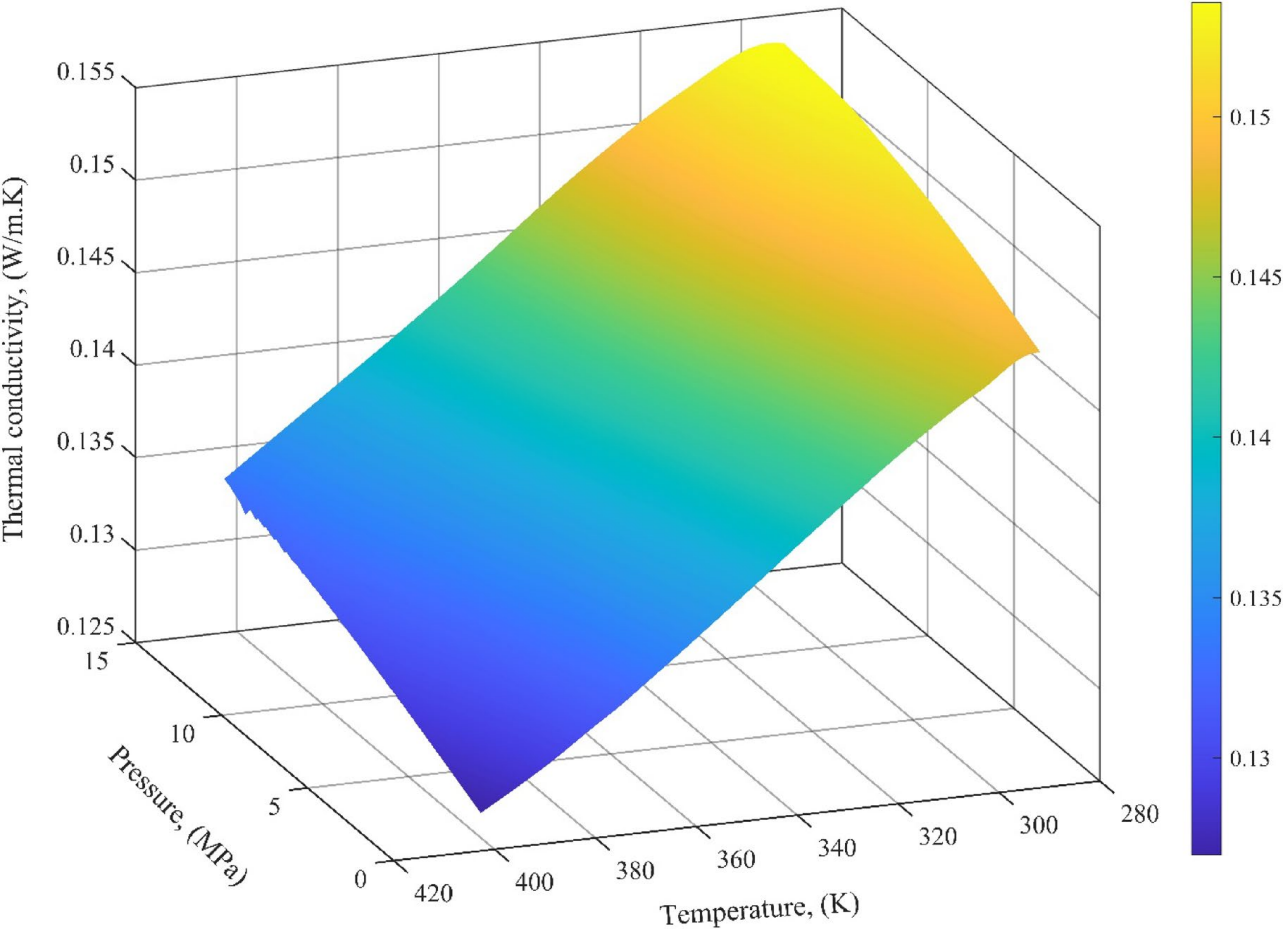
Simulated impact of pressure and temperature on LTC using the SVM model.

Figure 9 presents the simulated variation of LTC for methyl butyrate, a short-chain ester, as a function of pressure and temperature. The results align well with physical expectations and literature trends. As temperature increases, a clear decline in LTC is observed. This trend is attributed to enhanced molecular agitation at elevated temperatures, which disrupts the coherent energy transfer pathways in the liquid phase. The expansion of the fluid increases intermolecular distances, weakening the vibrational coupling responsible for thermal conduction. As a result, heat transfer becomes less efficient in less densely packed molecular systems. Conversely, increasing pressure leads to a notable rise in LTC. At higher pressures, molecules are forced into closer proximity, enhancing intermolecular interactions and reducing free volume. This compaction improves the propagation of energy through collisional and vibrational mechanisms. The positive correlation between pressure and LTC reflects the general behavior of liquids under compression, where thermal transport is facilitated by stronger short-range molecular forces. The model’s output not only aligns with experimental observations in similar systems but also reinforces the SVM model’s ability to capture the thermodynamic dependencies of LTC in ester biofuels.

**Fig. 10:**
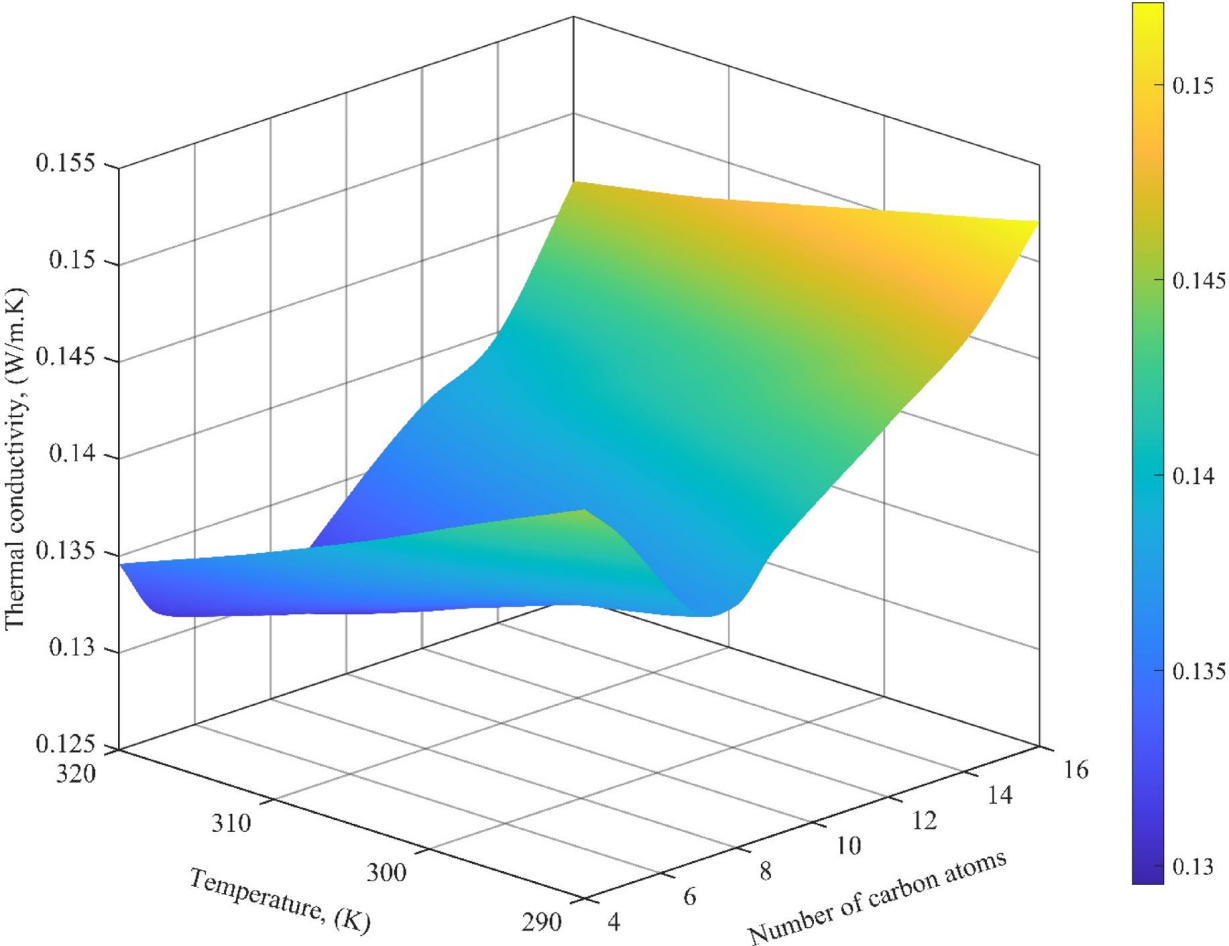
Simulated LTC values for FAEEs as a function of carbon chain length.

To explore the molecular structure–property relationship, Fig. 10 illustrates the LTC predictions for a series of FAEEs with varying carbon chain lengths. The results reveal a nonlinear trend: LTC initially decreases with increasing chain length up to eight carbon atoms, then gradually increases for longer-chain molecules. This pattern can be explained through competing physical mechanisms. For short-chain esters, the addition of carbon atoms leads to increased molecular complexity and reduced packing efficiency, which can disrupt heat conduction pathways, leading to a drop in LTC. However, beyond a certain chain length, the increase in van der Waals interactions and molecular alignment facilitates more effective energy transfer, thereby enhancing LTC. The carboxylic head group’s influence becomes comparatively less dominant in longer esters, and the extended hydrocarbon tail supports improved heat transfer through transient bonding and aligned motion. These results highlight the model’s capability to replicate subtle molecular-scale phenomena, further supporting its application for predictive screening of biofuel candidates with tailored thermal properties.

### Comparison against empirical correlations from literature

To provide a fair evaluation of the proposed LTC models, a comparison was conducted against several widely cited empirical correlations reported in the literature. Importantly, each correlation was only applied within its originally defined applicability domain (i.e., the same esters, temperature, and pressure ranges for which it was developed). For direct benchmarking, the SVM and GP models from this study were applied to the same restricted datasets, allowing one-to-one comparison under identical conditions. The results are summarized in Table 5. The empirical correlations, most of which are based on polynomial temperature–pressure regressions, show very low errors within their calibration ranges, often achieving MAPEs below 0.3%. However, these models typically rely on numerous adjustable coefficients (ranging from 9 to 48) and are limited to a small set of esters under narrow operating conditions. While this explains their high accuracy in those specific domains, it also highlights their limited transferability and need for recalibration when applied to other compounds or broader ranges. In contrast, the SVM and GP models developed here require only five general input features (temperature, pressure, molecular weight, critical pressure, and critical temperature) and do not depend on compound-specific fitting. Despite being trained on a much larger and more diverse dataset, the SVM model consistently reproduces the experimental datasets with MAPEs below 0.75%, and the GP correlation stays within 2% in all cases. This performance demonstrates that the proposed models not only compete with but also approximate the accuracy of specialized empirical correlations, while offering broader applicability and a simpler, more general framework. Thus, the comparison is not intended to claim that existing correlations are inaccurate; rather, it demonstrates that while those correlations are highly precise within narrow ranges, their usability is constrained. By contrast, the SVM and GP models achieve comparably low errors without re-calibration, across multiple esters and operating conditions, highlighting their superior generalization capability and practical value for engineering applications.

**Table 5:** Comparison of MAPE values reported by literature correlations for their respective datasets, alongside prediction errors from the SVM and GP models when applied to the same data.

Data reference	Ester biofuel analyzed	Empirical correlation	Quantity of data	Number of adjustable coefficients	MAPE value of correlation, (%)	MAPE of SVM, (%)	MAPE of GP, (%)
^33^	Methyl pelargonate, Methyl caprylate, Methyl caproate, Methyl pentanoate, Methyl butyrate	$\:\lambda\:=\sum\:_{i=0}^{2}{a}_{i}{T}^{i}$	99	15	0.17	0.42	0.86
^21^	Methyl caprate, Methyl laurate, Methyl myristate	$\:\lambda\:=\sum\:_{i=0}^{2}{a}_{i}{T}^{i}$	78	9	0.20	0.53	1.42
^22^	Ethyl heptanoate, Ethyl caprylate, Ethyl caprate, Ethyl laurate, Ethyl myristate	$\:\lambda\:=\sum\:_{i=0}^{2}{a}_{i}{T}^{i}$	77	15	0.21	0.71	1.82
^26^	Methyl caprate, Methyl laurate, Methyl myristate	Equation (1)	60	48	0.09	0.49	1.44
^32^	Ethyl hexanoate, Ethyl pentanoate, Ethyl butyrate, Ethyl propionate, Ethyl acetate	$\:\lambda\:=\sum\:_{i=0}^{2}{a}_{i}{T}^{i}$	96	15	0.21	0.57	1.98
^30^	Methyl Decanoate, Methyl Octanoate, Methyl Pentanoate	$$\begin{aligned}\:&\lambda\:=\sum\:_{i=0}^{3}\\&\:\sum\:_{j=0}^{1}{a}_{ij}{{T}_{red}}^{i}{{P}_{red}}^{j}\end{aligned}$$	328	24	0.28	0.62	1.26
^31^	Methyl caproate, Methyl butyrate	Equation (1)	158	18	0.40	0.65	1.59

### Sensitivity analysis of input features influencing LTC

To better understand the role of each variable in shaping the predicted values of LTC, a sensitivity analysis was conducted using the outputs of the SVM model, which demonstrated the highest overall accuracy in this study. This analysis quantifies how strongly each input feature correlates with the LTC predictions and helps prioritize the variables for future data collection and model refinement. The Pearson correlation coefficient was used as the statistical metric to assess linear relationships between LTC and the five model inputs:

16 $$\:R\left(k,X\right)=\frac{\sum\:_{i=1}^{n}\left({k}_{i}-\overline{{k}_{i}}\right)\left({k}_{i}-\overline{{k}_{i}}\right)}{\sqrt{\sum\:_{i=1}^{n}{\left({k}_{i}-\overline{{k}_{i}}\right)}^{2}\sum\:_{i=1}^{n}{\left({k}_{i}-\overline{{k}_{i}}\right)}^{2}}}$$

This factor ranges between − 1 and + 1, where positive values indicate direct correlations and negative values represent inverse relationships. The magnitude reflects the strength of association.

**Fig. 11:**
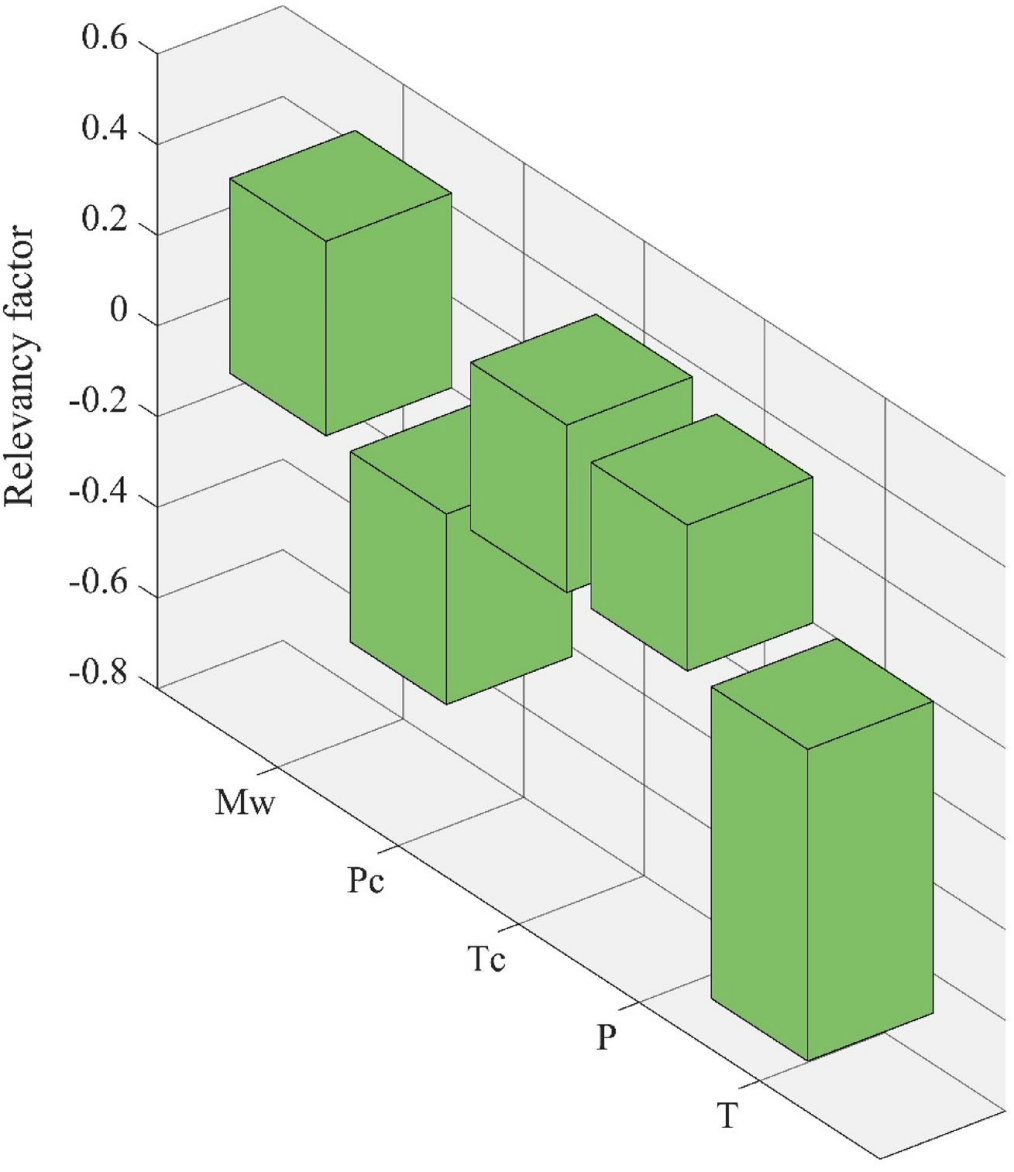
The relationship between LTC and each model input variable based on SVM model predictions.

The resulting correlation values are illustrated in Fig. 11. The analysis reveals that temperature exerts the most pronounced influence on LTC, with a strong negative correlation. This outcome aligns with the kinetic theory of liquids and free-volume theory, which describe how thermal conductivity in liquids is governed by intermolecular interactions and energy transfer through collisional and vibrational mechanisms. As temperature increases, molecules gain kinetic energy and vibrational amplitudes increase, disrupting the short-range order that facilitates coherent vibrational energy transfer. Simultaneously, thermal expansion increases the average intermolecular spacing, reducing collision frequency and weakening vibrational coupling. These combined effects explain why LTC decreases with increasing temperature, a behavior consistently observed in experimental studies of organic liquids.

The second most influential parameter is molecular weight, which shows a positive correlation with LTC. Larger ester molecules have extended hydrocarbon chains that increase the surface area for van der Waals interactions and enhance transient alignment between neighboring molecules. From the perspective of Eyring’s theory of liquid transport and mode-coupling approaches, heavier molecules also exhibit reduced translational mobility but stronger collective vibrational modes, which can facilitate more effective energy transfer. This explains why esters with higher molecular weight, despite lower diffusivity, tend to exhibit improved thermal conductivity compared to their lighter counterparts.

The critical temperature also contributes positively to LTC, reflecting the intrinsic stability and cohesive energy of a fluid. Compounds with higher T_c_ generally possess stronger intermolecular attractions, which enhance collisional pathways for energy transfer. By contrast, critical pressure displays a weaker and slightly negative correlation. While pressure does enhance density and intermolecular contact at a given state point, its influence is less dominant within the experimental range studied, since most liquids are already densely packed under ambient conditions. This is consistent with the hard-sphere and cell theories of liquids, which suggest that once a dense packing fraction is reached, additional compression has a diminishing effect on thermal transport.

Interestingly, operating pressure was found to have the weakest correlation among all variables. Although increasing pressure does increase LTC by reducing free volume and forcing molecules into closer proximity, the magnitude of this effect is relatively small compared to the strong disruptive influence of temperature and the structural contributions of molecular weight and T_c_. This suggests that within the studied range, LTC is governed primarily by molecular-scale energetics rather than macroscopic compression effects.

Overall, this analysis emphasizes that accurate LTC modeling for ester biofuels relies primarily on capturing the dominant temperature dependence, supported by reliable molecular descriptors such as molecular weight and critical temperature, which encode structural and cohesive properties. These findings not only corroborate the predictive trends observed in this study but are also consistent with established theoretical frameworks for liquid heat conduction, reinforcing the physical interpretability of the developed models.

### Limitations and future directions

A Despite the strong predictive accuracy and robustness demonstrated by the developed frameworks, certain limitations must be acknowledged. First, the models are trained exclusively on ester-based biofuels, and their direct applicability to other classes of renewable fuels (e.g., alcohols, ethers, or mixed biofuel blends) remains untested. Extrapolation beyond the established input domain, particularly outside the ranges reported in Table 2, may reduce reliability. Second, the present work relies on a dataset compiled from different experimental sources, each with unique uncertainties and variations in measurement techniques. While these uncertainties are relatively small (below 2%), systematic biases may still influence the learned relationships. Third, although the GP approach provides an explicit analytical correlation, its complexity grows with additional variables, and over-simplification during symbolic regression may obscure subtle nonlinear effects.

Future studies could extend this work in several directions. Expanding the dataset to include other biofuel derivatives and mixed-fuel systems would allow the models to generalize more broadly across renewable energy applications. Incorporating molecular descriptors derived from quantum chemical calculations or molecular dynamics simulations could further enhance the physical interpretability of the models. In addition, integrating uncertainty quantification and probabilistic machine learning methods would provide confidence intervals alongside deterministic predictions, supporting risk-aware engineering applications. From a practical standpoint, embedding the derived models into process simulators or engine performance codes could accelerate their adoption in real-world design and optimization workflows. Finally, as larger experimental databases become available, deep learning architectures and hybrid physics-informed ML approaches may offer even greater accuracy while preserving interpretability.

## Summary and conclusions

This study presented a comprehensive machine learning framework for predicting the LTC of ester biofuels using an extensive dataset of 1,641 experimental measurements covering 22 different methyl and ethyl esters across broad ranges of temperature and pressure. The predictive models were developed using SVM, DT, and GP techniques. Each model was trained and validated on a uniformly structured input space comprising temperature, pressure, critical properties, and molecular weight, allowing for generalizable and compound-independent LTC estimation. Among the models, the SVM demonstrated superior performance with a testing MAPE of 0.60% and narrow error distribution across both training and testing sets. The GP method successfully generated an explicit, easy-to-use mathematical correlation that also achieved high accuracy, with a MAPE of 1.16%, offering an interpretable alternative for real-time applications. Reliability assessments through statistical plots, error distributions, and outlier analysis confirmed the robustness and stability of all developed models. Furthermore, the models showed strong generalization when tested separately on FAMEs and FAEEs, highlighting their versatility across structurally diverse biofuels. Comparative benchmarking with existing empirical correlations from the literature revealed that the proposed methods not only maintain competitive accuracy but also eliminate the need for complex, case-specific formulations. The models accurately captured key LTC trends, including its decrease with temperature, increase with pressure, and nonlinear variation with carbon chain length, demonstrating strong capability to reflect both thermodynamic and molecular-level effects in ester biofuels. Sensitivity analysis underscored temperature as the most dominant factor influencing LTC, followed by molecular weight and critical thermodynamic properties, while pressure exhibited relatively minor impact.

Overall, the models developed in this work offer a reliable, generalizable, and high-fidelity approach for predicting LTC in ester biofuels, supporting their integration into process simulations, combustion modeling, and thermal design applications within renewable energy systems.

## Supplementary Information

Below is the link to the electronic supplementary material.

Supplementary Material 1

## Data Availability

The datasets used and/or analyzed during the current study available from the corresponding author on reasonable request.
